# Increased Expression of Flightless I in Cutaneous Squamous Cell Carcinoma Affects Wnt/β-Catenin Signaling Pathway

**DOI:** 10.3390/ijms222413203

**Published:** 2021-12-08

**Authors:** Gink N. Yang, Xanthe L. Strudwick, Claudine S. Bonder, Zlatko Kopecki, Allison J. Cowin

**Affiliations:** 1 Future Industries Institute, University of South Australia, Adelaide 5095, Australia; gink.yang@mymail.unisa.edu.au (G.N.Y.); xanthe.strudwick@unisa.edu.au (X.L.S.); zlatko.kopecki@unisa.edu.au (Z.K.); 2 Center for Cancer Biology, University of South Australia and SA Pathology, Adelaide 5000, Australia; claudine.bonder@unisa.edu.au; 3 Adelaide Medical School, University of Adelaide, Adelaide 5000, Australia; 4 Clinical and Health Sciences, University of South Australia, Adelaide 5000, Australia

**Keywords:** Flightless, squamous cell carcinoma, cancer metastasis, non-melanoma skin cancer

## Abstract

Cutaneous squamous cell carcinoma (cSCC) accounts for 25% of cutaneous malignancies diagnosed in Caucasian populations. Surgical removal in combination with radiation and chemotherapy are effective treatments for cSCC. Nevertheless, the aggressive metastatic forms of cSCC still have a relatively poor patient outcome. Studies have linked actin cytoskeletal dynamics and the Wnt/β-catenin signaling pathway as important modulators of cSCC pathogenesis. Previous studies have also shown that the actin-remodeling protein Flightless (Flii) is a negative regulator of cSCC. The aim of this study was to investigate if the functional effects of Flii on cSCC involve the Wnt/β-catenin signaling pathway. Flii knockdown was performed using siRNA in a human late stage aggressive metastatic cSCC cell line (MET-1) alongside analysis of *Flii* genetic murine models of 3-methylcholanthrene induced cSCC. Flii was increased in a MET-1 cSCC cell line and reducing Flii expression led to fewer PCNA positive cells and a concomitant reduction in cellular proliferation and symmetrical division. Knockdown of Flii led to decreased β-catenin and a decrease in the expression of the downstream effector of β-catenin signaling protein SOX9. 3-Methylcholanthrene (MCA)-induced cSCC in *Flii* overexpressing mice showed increased markers of cancer metastasis including talin and keratin-14 and a significant increase in SOX9 alongside a reduction in Flii associated protein (Flap-1). Taken together, this study demonstrates a role for Flii in regulating proteins involved in cSCC proliferation and tumor progression and suggests a potential role for Flii in aggressive metastatic cSCC.

## 1. Introduction

The incidence of cutaneous squamous cell carcinoma (cSCC) has been rising at an alarming rate in the last three decades, with higher numbers of aggressive metastatic tumors observed in men, the elderly and in immunosuppressed patients [1]. Identifying new target proteins that regulate metastatic cell division, proliferation and signaling pathways critical to cancer cell progression and invasion may aid the development of therapeutic approaches to reduce the burden on aggressive metastatic cSCC in the community. Dynamic actin remodeling has been shown to underpin cancer cell division, proliferation and epithelial–mesenchymal transition [2]. Several studies have shown that dysregulation of the actin cytoskeleton in tumor cells is linked to aberrant tumor–microenvironment interactions that promote invasion and metastasis of aggressive cSCC [3,4,5]. Additionally, expression of actin remodeling proteins has been shown to change during tumor development and therapies that target these proteins may inhibit cell motility and invasion [6,7,8].

Flightless I (Flii), a multifunctional actin remodeling protein with both intracellular and extracellular roles, has been shown to modulate cellular functions and signaling pathways critical in wound repair, inflammation and cancer progression [9,10,11]. Flii expression is increased during development and tissue repair and its levels can vary in different tumor types, such as breast, prostate and colorectal cancers, with high Flii levels being linked to negative patient prognosis [11,12,13]. Factors which affect the level of Flii have yet to be fully identified; however, Flii has been shown to have several binding partners which regulate actin dynamics (e.g., paxillin, talin), transcription and translation (e.g., LRRFIP1, Akt, Ulk1) and inflammation (e.g., LPS, caspase-1, LRRFIP2, Myd88) via signaling pathways important during cancer progression [14]. Flii intracellular roles have been linked to its function in cellular adhesion and migration [10,15], collagen remodeling [16,17,18], nuclear receptor co-activation promotion of transcriptional activity associated with different cancer cell lines [11,12], hormone receptor regulation of lipid metabolism [19], chromatic remodeling [20] and osteogenic differentiation [21]. A number of studies have shown both beneficial and detrimental effects of Flii in cancer progression (for a detailed review see [14]); however, in the context of human cSCC, increased Flii within invading cells at the tumor edge and overexpression in a mouse model of cSCC lead to significantly larger, more necrotic tumors due to decreased apoptosis and increased invasion of tumor cells [22]. In contrast, reducing Flii results in lower levels of invasion of human cSCC cell lines in vitro and decreased development and progression of cSCC tumors in mice in vivo [22]. Additionally, Flii association with Leucine Rich Repeat Flightless Interacting Protein 1 (LRRFIP1/Flap-1) has been linked to regulation of epithelial–mesenchymal transition through regulation of the Wnt/β-catenin signaling pathway, a process strongly linked to invasion of aggressive cSCC [23,24]. Flii may regulate cancer progression through its effects on proteins involved in nuclear export and subsequent mRNA translation [20]. Furthermore, Flii affects cell polarity, progenitor cell division and epidermal differentiation and stratification during development and repair [25,26,27,28]. Taken together, Flii effects on cellular processes and signaling pathways may provide better understanding of the development and progression of aggressive metastatic cSCC. 

The objective of this study was to investigate the effect of altering the level of Flii in an aggressive metastatic cSCC cell line (MET-1) to better understand its functional effect on cellular processes critical in cSCC development and progression, including cell division, proliferation, differentiation and Wnt/β-catenin signaling. 

## 2. Materials and Methods

### 2.1. Cell Culture

Human immortalized keratinocytes (HaCaTs, ATCC, Manassas, VA, USA) were cultured in low glucose DMEM medium (Thermo Fisher Scientific, Melbourne, Australia) with 2 mM L-glutamine, 10% FBS (Thermo Fisher Scientific, Melbourne, Australia) and 100 U/mL penicillin/streptomycin (Lonza, Basel, Switzerland). Late-stage human cSCC cell line MET-1 was originally collected from an invasive primary tumor from the forehead skin of an adult immunosuppressed patient and gifted by Professor Charlotte Proby from the University of Dundee 29. MET-1 cell line was cultured following previously established protocols (22).

### 2.2. Animal Studies

All experiments and maintenance of mice were conducted in accordance with the “Australian Code of Practice for the Care and Use of Animals for Scientific Purpose”, using protocols approved by the Women’s and Children’s Health Network Animal Ethics Committee (WCHN) (AEC 909/01/15). All mouse strains were congenic on the BALB/c background. *Flii* deficient mice were generated by switching a null allele (*Flii*^tm1Hdc^) with an endogenous allele (*Flii^+^*) locus, with animals heterozygous for this mutation designated *Flii^+/−^* [29]. WT (wild-type) littermates to *Flii^+/−^* mice used as WT control animals. Transgenic *Flii* overexpressing mice (strain name: (Tg1FLII) 2Hdc) were generated by incorporating a 17.8-Kb fragment of a human cosmid clone that spans the entire *FLII* locus, with animals homozygous for the transgene in addition to the endogenous *Flii* allele designated *Flii^Tg/Tg^*. Details regarding the generation of the transgenic mice strains were described previously, showing elevated levels of Flii protein in various tissues including skin [30]. 

### 2.3. Mouse Model of Cutaneous Squamous Cell Carcinoma

A primary invasive cSCC tumor mouse model was used in 12-week old female *Flii^+/−^*, WT and *Flii^Tg/Tg^* BALB/c mice as described in [22]. Briefly, cSCC was induced by a single intradermal injection of carcinogen 3-Methylcholanthrene (MCA, Sigma-Aldrich, Sydney, Australia) at a dosage of 500 μg MCA (pre-dissolved in 100 μL of corn oil at 37 °C) per animal. Necrotic and ulcerated cSSC developed over a 12-week period. Mice were humanely killed and tumor skin samples harvested at week 12. Tumor invasion and tumor size was confirmed using Hematoxylin and Eosin and pan-cytokeratin staining as previously described [22]. Power studies showed that a sample size of 12 would give 90% power using a 5% test level and a one-tailed test. A total of 12 mice per genotype per time point were used for the experiments.

### 2.4. Immunofluorescent Analysis of In Vivo cSSCs

Paraffin-embedded tumor samples were sectioned (4 μm) prior to antigen retrieval using citrate buffer and trypsin as described previously [31]. Following antigen retrieval, 3% normal goat serum diluted in phosphate-buffered saline was used for blocking for 30 min. Primary antibodies including Flii (1:200, Santa Cruz Biotechnology, Sydney, Australia), Flap-1/LRRFIP1 (1:400, Bioss, Woburn, USA), PCNA (1:200, Santa Cruz Biotechnology, Sydney, Australia), K1 (1:200, Abcam, Sydney, Australia), K14 (1:100, Abcam, Sydney, Australia), β-catenin (1:200, Santa Cruz Biotechnology, Sydney, Australia), SOX9 (1:1000, Abcam, Sydney, Australia), BrdU (1:500, Sigma-Aldrich, Sydney, Australia), pH3(s28) (1:400, Abcam, Sydney, Australia), γ-tubulin (1:500, Abcam, Sydney, Australia), Talin (1:500, Millipore, Sydney, Australia) were diluted in blocking buffer and applied. Species-specific Alexa Fluor 488, 568 or 633-conjugated secondary antibodies (1:400, Invitrogen, Melbourne, Australia) were diluted in phosphate-buffered saline and applied for detection. For detection of actin, directly conjugated Oregon Green 488 Phalloidin (1:400, Thermo Fisher, Melbourne, Australia) antibody was used. Nuclear counterstain 4,6-diamidino-2-phenyindole (DAPI) (1:5000) was applied last. 

### 2.5. siRNA Knockdown of Flii

Flii siRNA knockdown was performed in late-stage human cSCC cell line MET-1, which was originally collected from an invasive primary tumor from the forehead skin of an adult immunosuppressed patient and gifted by Professor Charlotte Proby from the University of Dundee [32]. Protocols used for the Flii knockdown were optimized as previously described [33]. Briefly, MET-1 cells were cultured in antibiotic- and serum-free MET-1 media overnight to synchronize the cell cycle. On the day of transfection, cells were trypsinized and centrifuged for 5 min at 1500× *g*. Cells were washed twice in sterile PBS and viable cell numbers counted using trypan blue exclusion in a hemocytometer. Knockdown solutions were prepared as follows: 50 μL per well of Opti-MEM (Life Technologies, Carlsbad, CA, USA) was combined with 0.5 μL per well of Lipofectamine 2000 (Thermo Fisher, Melbourne, Australia) and incubated for 30 min at room temperature. In parallel, 50 μL per well of Opti-MEM was combined with 0.5 μL per well of siRNA (siFlii 5′-GCUGGAACACUUGUCUGUGTT-3′ and 5′-CACAGACAAGUGUUCCAGCTT-3′, siControl 5′-GGUUAGCCGCACGUUAGUUTT-3′ and 5′-AACUAACGUGCGGCUAACCTT-3′, Sigma-Aldrich, Sydney, Australia) and incubated for 30 min at room temperature. The final knockdown solution was achieved by combining these two Opti-MEM-based solutions and further incubation for 30 min at room temperature. Triplicates of 1 × 10^5^ MET-1 cells per well for each time point assessment in a well of a 24-well plate were seeded in the final knockdown solution and incubated for 12 h at 37 °C, 5% CO_2_. Cells were given fresh Opti-MEM media containing 20% FBS at the end of 12 h and refreshed every 24 h. Confirmation of Flii knockdown was demonstrated using Western Blotting for samples collected at 24, 48 and 72 h. Knockdown cells were trypsinized and re-pelleted in appropriate media for other assays. 

### 2.6. WST-1 Proliferation Assay

Cell proliferation assays were performed on MET-1 cells following Flii siRNA knockdown according to the manufacturer’s protocols (Roche Applied Science, Sydney, Australia) and previously described protocols [33]. Cells were counted using a hemocytometer, and 6 replicates of 5000 cells per well for each treatment were seeded in a well of a 96-well plate in serum free MET-1 media overnight to synchronize cell cycle. On the day of experiment, cells were changed back to MET-1 media containing 20% FBS and 10 μL of WST-1 reagent (Roche Applied Science, Sydney, Australia) was added to each well, followed by a brief mixing via tapping the plate. Negative controls consisted of SiControl, cells with media only and another with no cells. The plates were incubated at a time interval of 24 and 48 h at 37 °C in 5% CO_2_. At each time point, the plate was read in a dual absorbance detector (at 460 and 620 nm) to measure the level of cell metabolic activity indicated by the amount of formazan dye produced during WST-1 enzymatic reaction. The read-out from experimental wells was blanked with the media-only control readings.

### 2.7. Protein Isolation and Western Blot

Following brief homogenization in lysis buffer (50 mM Tris pH 7.5, 1 mM EDTA, 50 mM NaCl, 0.5% Triton X-100) containing protease inhibitor tablet (1 per 10 mL; Complete, Mini (Roche Applied Science, Sydney, Australia)), samples were centrifuged and supernatants collected. A total of 50 µg of protein was run on 10% SDS-PAGE gels at 100 V for 1 h and transferred to nitrocellulose membrane using standard Towbin Buffer with 20% Methanol at 100 V for 1 h. Following blocking in 15% milk-blocking buffer for 10 min. Primary antibodies including Flii (1:200, Santa Cruz Biotechnology, Melbourne, Australia), β-catenin (1:200, Santa Cruz Biotechnology, Melbourne, Australia), β-tubulin, Axin2 and SOX9 (1:300, Abcam, Sydney, Australia) were diluted in buffer and applied to the membrane at 4 °C overnight. Species-specific secondary horseradish peroxidase-conjugated antibodies were diluted in 5% milk-blocking buffer and applied to the membrane at room temperature for 1 h. Protein bands were detected using SuperSignal West Femto (Pierce Biotechnology, Melbourne, Australia) and visualized with GeneSys analysis software (Syngene, Brisbane, Australia).

### 2.8. BrdU-Cytochalasin D Pulse-Chase Labeling of Flii siRNA Cells

MET-1 cells +/− Flii siRNA were plated on glass coverslips pre-coated with fibronectin and collagen (50 μg/mL, Invitrogen) in the wells of a 6-well plate at a density of 20,000 cells in Epilife medium containing 10 μM BrdU (Sigma-Aldrich, Sydney, Australia) [34]. BrdU was allowed to incorporate into the DNA synthesis (pulse-process) for 24 h followed by washing in sterile PBS twice. New Epilife medium containing 5 μM cytochalasin D (cytD) was then added to the cells to arrest cytokinesis (chase-process) for 24 h, followed by washing in sterile PBS twice. The cells were subsequently washed twice in washing buffer (0.5% BSA in PBS) and fixed in 4% paraformaldehyde. Fixed cells were permeabilized in buffer (0.2% Triton X-100 in washing buffer) followed by blocking in washing buffer. Primary antibodies including BrdU (1:400, Sigma-Aldrich, Sydney, Australia) and pH3(s28) (1:400, Abcam, Sydney, Australia) were diluted in washing buffer and applied. Species-specific Alexa Fluor 568 or 633-conjugated secondary antibodies (1:400, Invitrogen, Melbourne, Australia) were diluted in washing buffer and applied along with Oregon Green 488 Phalloidin (1:400, Thermo Fisher Scientific, Melbourne, Australia) and DAPI (1:5000). Stained cells were imaged and positive cells counted using Olympus cellSens Dimension software. 

### 2.9. Statistical Analyses

Statistical differences for in vitro siRNA studies and in vivo immunofluorescence studies were determined using one-way ANOVA for multiple comparisons. Statistical differences for comparison between two genotypes were determined using two tailed and equal variance Student’s t-test. Statistical differences for in vitro protein expression studies involving only two groups of cells were determined using a one-tailed and unequal variance Student’s t-test. *p* < 0.05 was considered statistically significant. *p* < 0.005 was considered highly significant.

## 3. Results

### 3.1. Flii Levels Are Increased in Human Metastatic cSCC Cells and Correlate with Increased Wnt/β-Catenin Signaling and Cytoplasmic SOX9 Expression

**Figure 1:**
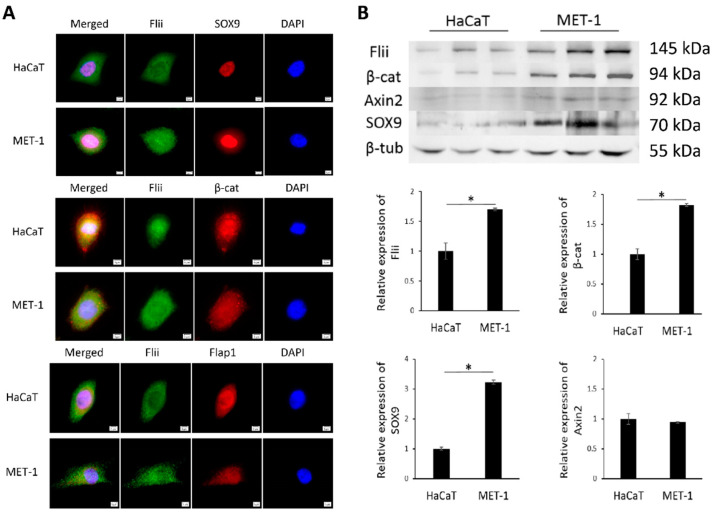
Flii is increased in human late stage metastatic cSCC cell line and colocalizes with Wnt/β-catenin signaling pathway molecules. (**A**) Dual immunocytochemistry of Flii (green) and SOX9, β-catenin (β-cat) and Flap1 (red) in HaCaT and MET-1 cells. Nuclear DAPI staining shown as pseudo-stained blue. Scale bars, 5 μm. (**B**) Western blot analysis of Flii, β-catenin, Axin2, SOX9 and β-tubulin protein levels in HaCaT and MET-1 cells. Equal amount of cell lysate (50 μg) was loaded in each lane. Quantification of relative protein levels was achieved using densitometry after normalizing to β-tubulin. Mean ± SEM. n = 3. * *p* < 0.05.

We first investigated the localization and expression of Flii, β-catenin, Axin2, Flap-1 and SOX9 in immortalized human keratinocytes (HaCaT) and tumorigenic MET-1 cell line established from a patient with late-stage aggressive metastatic cSCC [22,32]. Immunocytochemistry showed that Flii was observed in the nucleus and cytoplasm of both HaCaT and MET-1 cell lines (Figure 1A). Western blot analysis showed significantly higher Flii levels in a tumorigenic MET-1 cSCC cell line compared to HaCaTs (Figure 1B). Flii colocalized with nuclear SOX9 and both cytoplasmic and nuclear β-catenin in human cSCC cell line, suggesting involvement in Wnt/β-catenin signaling. No apparent difference in localization of β-catenin was observed between the two cell lines; however, like Flii, significantly higher levels of β-catenin were also observed in a MET-1 cSCC cell line (Figure 1A,B). 

Flap-1 has previously been linked to regulation of epithelial–mesenchymal transition via the Wnt/β-catenin signaling pathway, which is critical for the invasion of aggressive cSCC [24]. Like Flii, localization of Flap-1 was also detected in both the nucleus and cytoplasm, with no apparent differences between the non-tumorigenic and tumorigenic cell lines (Figure 1A). SOX9 expression was also observed in the nuclei of HaCaT and MET-1 cells; however, only MET-1 cSCC cells showed cytoplasmic expression of SOX9 (Figure 1A). Additionally, like Flii, total levels of SOX9 were significantly increased in a tumorigenic MET-1 cSCC cell line compared to HaCaT cell line (Figure 1B). Axin2 total protein levels did not differ between HaCaT and MET-1 cell lines. 

### 3.2. Flii Knockdown Decreases Proliferation, Division and Wnt/β-Catenin Signaling in Human Metastatic cSCC

**Figure 2:**
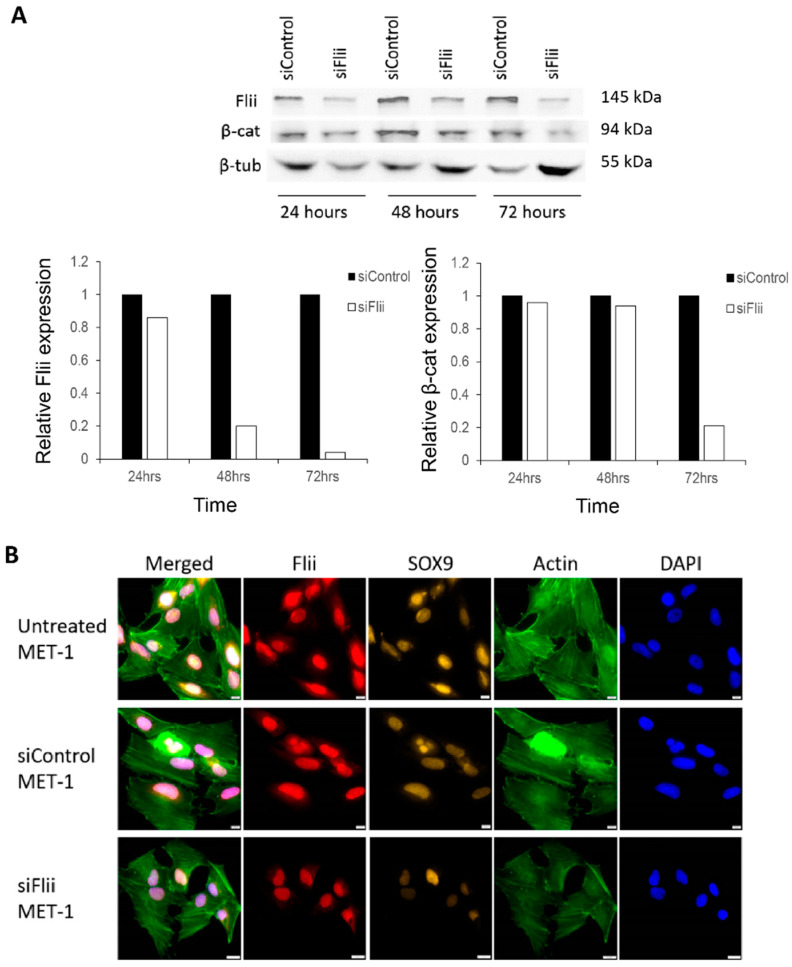
Knockdown of Flii in human cSCC cell line reduces SOX9 but not β-catenin levels. (**A**) Western blot analysis and graphical quantification of Flii, β-catenin and β-tubulin protein levels in MET-1 cells treated with siControl and siFlii at 24, 48 and 72 h intervals. Samples are pooled from three independent technical repeats. (**B**) Immunocytochemistry of Flii (red), SOX9 (yellow), Actin (green) and DAPI (blue) in MET-1 cells following Flii knockdown. Scale bars, 5 μm.

We next addressed if reducing *Flii* expression could alter the functional characteristics associated with aggressive metastatic cSCC. Specifically, Flii expression was transiently reduced via lipofectamine-based siRNA knockdown in human cSCC MET-1 cells with Western blot analysis used to confirm stable knockdown of Flii expression for up to 72 h (Figure 2A). At 48 h post siRNA knockdown, siFlii-MET-1 cells showed reduced immunofluorescence of Flii when compared to untreated and siControl-MET-1 cells (Figure 2B). Concurrently, cytoplasmic SOX9 expression was also reduced in siFlii-MET-1 cells when compared to untreated and siControl-MET-1 cells (Figure 2B). Consistently, reduction in the distribution of filamentous actin stained with phalloidin was also observed in siFlii-MET-1 cells when compared to untreated and siControl-MET-1 cells (Figure 2B), a known functional consequence of reduced Flii expression [15].

**Figure 3:**
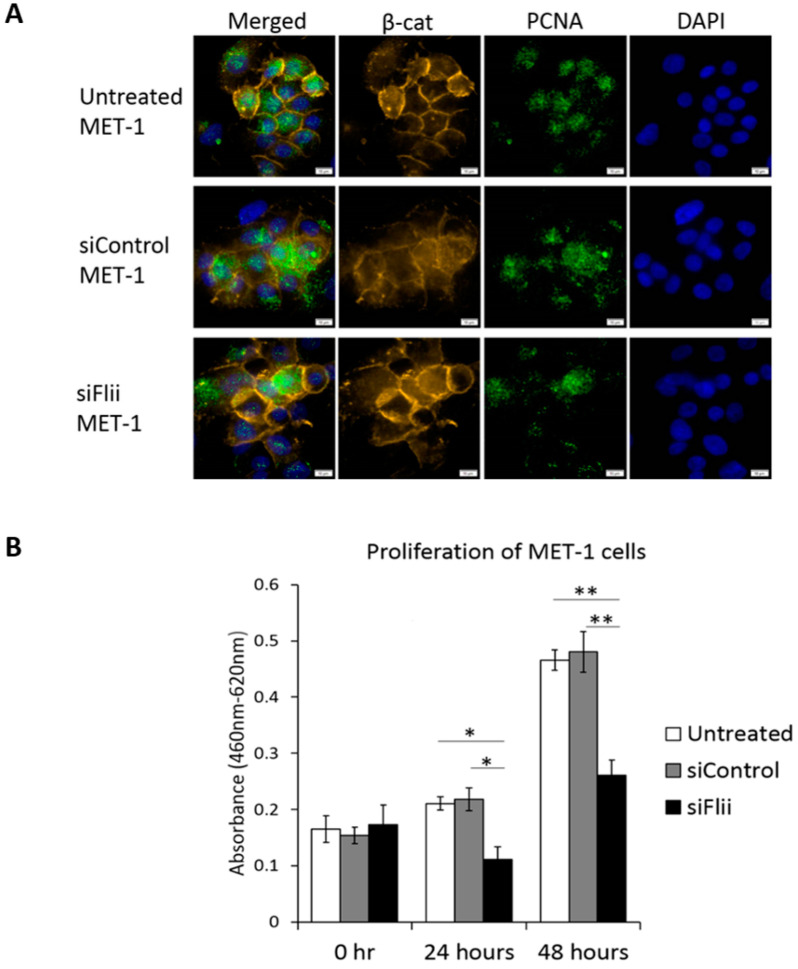
Knockdown of Flii in human cSCC cell line leads to decreased cell proliferation independent of β-catenin. (**A**) Immunocytochemistry of β-catenin (yellow), PCNA (green) and DAPI (blue) in MET-1 cells following Flii knockdown showing no effect on β-catenin and reduced expression of PCNA. Scale bars, 5 μm. (**B**) Quantification of WST-1 proliferation assay following Flii knockdown in MET-1 cells at 24 and 48 h showing significant reduction in cell proliferation. Mean ± SEM. n = 6. * *p* < 0.05, ** *p* < 0.005.

To determine the effect of Flii on proliferation, levels of proliferating cell nuclear antigen (PCNA) were assessed and compared to controls (Figure 3A). A clear reduction in PCNA was observed (Figure 3A,B). No apparent difference in expression of β-catenin levels was observed following Flii knockdown in a MET-1 cSCC cell line at this time point (48 h), despite an observed reduction in β-catenin level at 72 h (Figure 2A), suggesting a temporal effect. The functional consequence of Flii knockdown on proliferation of the MET-1 cSCC cell line was confirmed using a WST-1 proliferation assay, with significantly reduced proliferation (~50% reduction) of siFlii-MET-1 cells at both 24 and 48 h (Figure 3B). 

**Figure 4:**
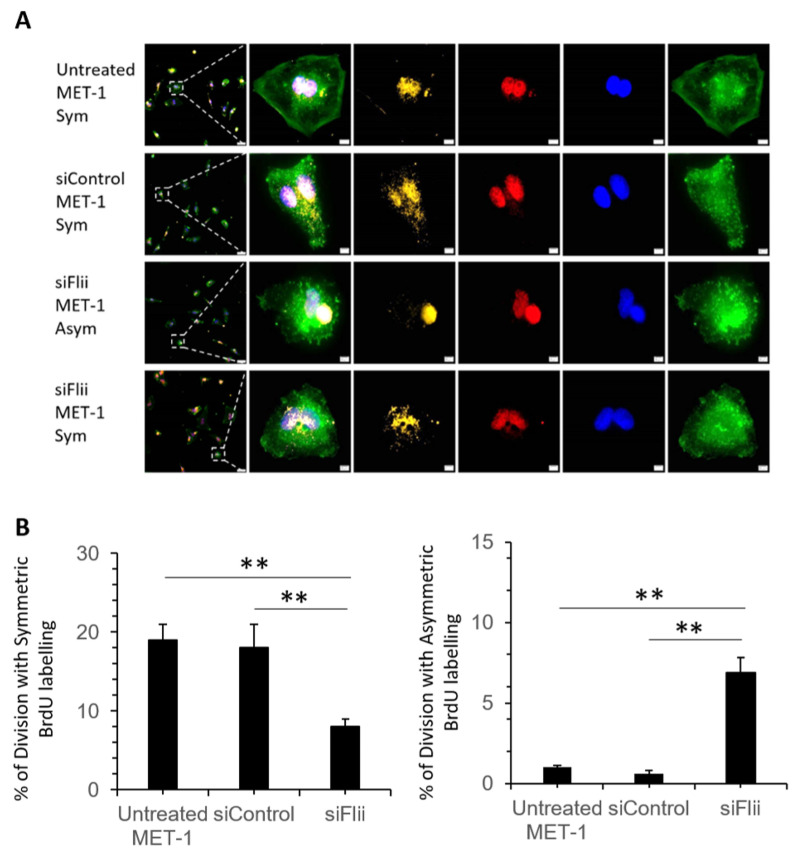
Effect of Flii knockdown on cell division pattern of human cSCC cell line. (**A**) Representative images of MET-1 cells (untreated), siControl-MET-1, siFlii-MET-1 cells undergoing symmetric (Sym) or asymmetric (Asym) division following siRNA knockdown and BrdU-cytD pulse-chase labeling with a total time of 48 h. Triple immunofluorescent staining of BrdU (yellow) showing DNA synthesis in proliferating cell, pH3 (red) identifying mitotic chromosomes during metaphase of proliferation and phalloidin (green) identifying actin filaments and nuclear DAPI (blue) staining. Asymmetrically dividing cells show skewed inheritance of BrdU-labeled DNA at the end of metaphase and symmetrically dividing cells show equal inheritance of BrdU-labeled DNA at the end of metaphase. Arrest of cytokinesis by cytD is confirmed by phalloidin staining showing disrupted formation of actin filaments in granular shape. Scale bars, 5 μm. (**B**) Quantification of mitotic cells undergoing symmetric or asymmetric division following BrdU-labeling, illustrating a decreased symmetric division and increased asymmetric division following Flii knockdown. Data represent the mean from three independent experiments with an average of 30 mitotic events per group. Mean ± SEM. n = 30. ** *p* < 0.005.

To examine if Flii knockdown affects cell division in human metastatic cSCC, a Bromodeoxyuridine-Cytochalasin D (BrdU-cytD) pulse-chase assay was carried out using MET-1 cells following siRNA knockdown of Flii. MET-1 cells have previously been shown to be poorly differentiated, express high levels of Keratin 14 marker and divide via symmetric cell division [32]. Globous pattern of actin was observed in all cytD treated cells, confirming actin disassembly at metaphase (Figure 4A). Flii knockdown by siFlii resulted in significantly decreased numbers of cells undergoing symmetric division and concurrent increase in number of cells undergoing asymmetric division as assessed via BrdU labeling in a human MET-1 cell line (Figure 4A,B).

### 3.3. Overexpression of Flii Promotes Metastatic Progression of Murine cSCC

**Figure 5:**
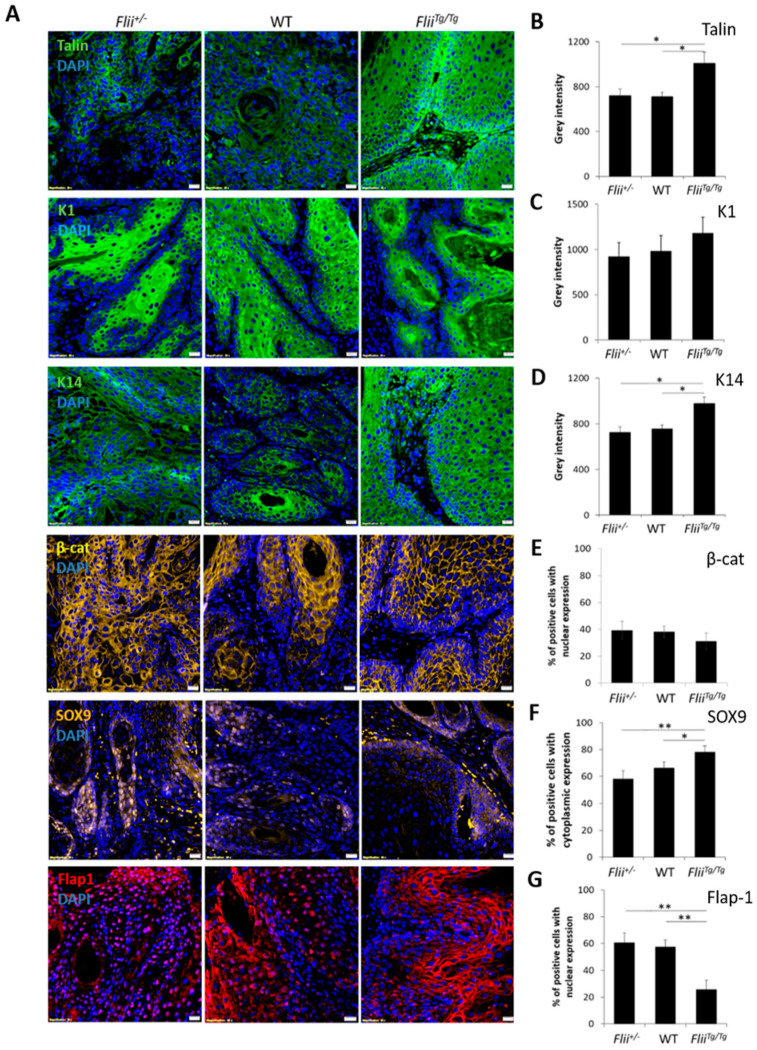
Overexpression of Flii promotes a poorly differentiated phonotype of tumorigenesis in cSCC. Immunofluorescence and quantification of talin (green) (**A**,**B**), K1 (green) (**A**,**C**), K14 (green) (**A**,**D**), β-catenin (yellow) (**A**,**E**), SOX9 (yellow) (**A**,**F**), Flap1 (red) (**A**,**G**) and DAPI (blue) (**A**) in sections of murine cSCC tumors of *Flii^+/−^*, wild-type and *Flii^Tg/Tg^* mice. Scale bars, 20 μm. Quantification of staining reveals increased talin, K14 and cytoplasmic SOX9 expression in *Flii^Tg/Tg^* mice with concurrent decrease in nuclear Flap1 expression suggestive of poorly differentiated tumors. Two-tailed paired Student’s t-test. Mean ± SEM. n = 10. * *p* < 0.05, ** *p* < 0.005.

To investigate the effect of altered Flii expression on metastatic progression of cSCC, in vivo cSCC tumors were induced in *Flii^+/−^*, wild-type and *Flii^Tg/Tg^* mice via intradermal injection of 3-Methylcholanthrene (MTA). Consistent with the previous finding showing that *Flii* overexpression resulted in more severe and aggressive cSCC development [22], cSCC tumors in *Flii^Tg/Tg^* mice showed significantly increased expression of Talin, an important marker of SCC cells with poorly differentiated morphology [35], when compared to *Flii^+/−^* and WT counterparts (Figure 5A,B). The effect of Flii on cSCC differentiation was examined by measuring the expression of K1 and K14 in keratin pearls of cSCC tumors. While there was no difference in expression of K1 between cSCC tumors in Flii genetic mice, overexpression of Flii resulted in significantly increased levels of K14, a known marker of poorly differentiated keratinocytes and late-stage metastatic cSCC cells [32], in keratin pearls of cSCC tumors of *Flii^Tg/Tg^* mice (Figure 5A,C,D), suggesting Flii promotion of metastatic progression in murine cSCC.

cSCC tumors in *Flii^+/−^*, wild-type and *Flii^Tg/Tg^* mice were also examined for β-catenin, SOX9 and Flap-1 expression. While no difference was observed in expression levels of β-catenin, Flii overexpression resulted in significantly increased SOX9 levels in cSCC tumors of *Flii^Tg/Tg^* mice compared to *Flii^+/−^* and wild-type counterparts (Figure 5A,E,F). SOX9 is a downstream effector of β-catenin activated transcription and a promoter of symmetric cell division during SCC metastasis [36]. *Flii^Tg/Tg^* cSCC tumors also showed a significant decrease in Flap-1 expression (Figure 5A,G). This suggests a possible interplay between Flii and Flap-1 during cSCC development, as Flap-1 is a naturally occurring antagonist of Flii previously shown to promote asymmetric cancer cell division [37] and regulate epithelial–mesenchymal transition in aggressive metastatic cSCC [23]. 

## 4. Discussion

Metastatic transformation of cSCC results in the development of aggressive tumors that are greatly dependent on complex re-organization of the cytoskeletal structures that regulate cell division and proliferation [1]. A number of genomic and transcriptomic studies have revealed a direct relationship between aberrant Wnt/β-catenin signaling and the aggressive metastatic nature of cSCC [38]. However, key molecular regulators that control the mechanisms that cause changes in the behavior of cSCC cells are still under investigation. Due to its central role in cytoskeletal regulation, Flii has been implicated in a range of cellular processes that require the modification of cell polarity, adhesion and contraction [15,18,25]. Flii has previously been shown to inhibit apoptosis and enhance tumor cell invasion, promoting cSCC progression in mice; however, the signaling pathways underpinning these findings have not been identified [22]. Flii has previously been shown to be present at high levels in cells from recurrent and metastatic cSCC from the same patient [22]. Here, we show significantly elevated Flii levels in a human cSCC cell line which colocalized with nuclear SOX9 and cytoplasmic and nuclear β-catenin, both of which were also elevated in terms of expression. This suggests that Flii may interact with Wnt/β-catenin signaling and effector proteins that can influence cSCC epithelial–mesenchymal transition during cancer invasion and metastasis. Unlike the previous studies which showed increased Axin2 signaling in response to increased Flii levels during ulcerative colitis [39], here we saw no difference in Axin2 levels in the cSCC cell line, suggesting a potential cell-dependent effect of Flii on Wnt/β-catenin signaling. 

Flii knockdown in the human aggressive metastatic cSCC cell line decreased cell proliferation with underlying effects on cell division and Wnt/β-catenin signaling. Flii knockdown led to decreased β-catenin expression in the human cSCC cell line and both nuclear and cytoplasmic expression of one of the main effector molecules, SOX9, was significantly reduced. SOX factors can act as feedback regulators that fine-tune Wnt/β-catenin signaling [40]. In addition to altering proliferation and symmetrical division to promote differentiation, reduction in Flii may further reduce tumor progression, whereby the exclusive nuclear SOX9 expression may increase the degradation of stabilized β-catenin. This agrees with the reduced β-catenin level observed following the loss of cytoplasmic SOX9. Therefore, together, these findings suggest that Flii effects on SOX9 expression in human cSCC may either promote degradation of stabilized β-catenin in the cytoplasm or act via a non-canonical β-catenin independent Wnt signaling pathway. 

Flii overexpression decreases epidermal stratification and affects epidermal cell division via Wnt/β-cat signaling [10,26,28]. We also found that knockdown of Flii in the human cSCC cell line reduced proliferation and decreased symmetric division and increased asymmetric division of the human MET-1 cSCC cell line. Symmetric division is increased in late-stage cancers and has been shown to promote undifferentiated proliferation of cancer cells, giving rise to a high degree of cellular heterogeneity in human cSCC, varying from well-differentiated to poorly differentiated tumors which present a higher rate of reoccurrence and lower rate of cure after treatment [41]. During SCC malignancy, cancer cells no longer differentiate and only proliferate in a replicating manner by symmetrical division, which results in increased tumor invasion and metastasis [42]. Consistent with this, increased expression of markers associated with poor differentiation and increased epithelial–mesenchymal transition and cancer metastasis in cSCC was observed in cSCC tumors of *Flii^Tg/Tg^* mice, including increased talin, K14 and cytoplasmic SOX9. Alteration of *Flii* gene expression in murine cSCC had minimal effect on expression of K1 in keratin pearls of cSCC tumors. Flii can interact directly with talin, contributing to altered integrin mediated signaling and cell adhesion [10], and high talin expression in cSCC of *Flii^Tg/Tg^* mice suggests that Flii may increase talin expression to promote increased activation of integrin-mediated pathways which are important during cSCC development. Talin has also been identified as the most highly expressed integrin–cytoskeleton crosslinker that promotes integrin activation and is associated with increased invasion of cSCC into the adjacent tissue [35]. Talin has also been shown to affect hemidesmosomal-like anchorage of keratinocytes and influence keratin network organization, including keratin filament bundling and nucleation in epithelial cancer cells [43]. Data presented here suggest that Flii may increase talin expression in cSCC to upregulate K14 signaling; however, further research is required to understand the exact mechanism. Our data have shown that high Flii levels may also promote increased symmetrical division of cancer cells, with high K14 expression in cSCC tumors, and this may contribute to increased tumor development as evident in cSCC of *Flii^Tg/Tg^* mice [22]. This is consistent with previous findings showing increased K14 expression and promotion of symmetrical division in *Flii^Tg/Tg^* mice during embryonic development [28]. Indeed, K14 had been identified as an important prognostic factor of cSCC tumor progression, where high K14 expression in the spinous and granular layers of human actinic keratosis is associated with an increased rate of cSCC development [44]. While studies, to date, have not demonstrated a direct link between Flii levels and cancer stage, these results suggest that Flii may be linked to more aggressive metastatic cSCC. 

Previous studies have demonstrated a clear link between symmetrically dividing cancer cells and increased cancer invasion due to increased epithelial to mesenchymal transition [45]. To investigate the role of altered Flii expression in epithelial to mesenchymal transition during cSCC development, we examined the levels of cytoplasmic and nuclear expression of SOX9 and Flap1 in keratin pearls of cSCC in *Flii^+/−^*, wild-type and *Flii^Tg/Tg^* mice. High Flii levels in cSCC tumors of *Flii^Tg/Tg^* mice resulted in a significantly increased number of cells with cytoplasmic SOX9 staining and significantly decreased number of cells with nuclear expression of Flap1, suggestive of increased epithelial to mesenchymal transition. The interplay between Flii and Flap-1 has previously been shown to inhibit transcription of β-catenin and TCF/LEF signaling, suggesting a role for Flii as a negative inhibitor of canonical Wnt signaling [46]. Meanwhile, silencing of Flap-1 in cancer cells has been linked to upregulation of β-catenin phosphorylation and decreased nuclear localization by the targeting of β-catenin destruction complex [24]. While the current study suggests a role for Flii in regulating epithelial to mesenchymal transition of keratinocytes, to further elucidate the role of Flii in cSCC metastasis, use of MET-1 cell lines in future studies with murine metastasis models in immunocompromised or syngeneic mice are required. Additionally, experiments could be undertaken assessing the metastatic rate of cSCC cells following Flii knockdown and subsequent effects on expression of stem-like genes important in driving cSCC metastasis, including OCT4, SOX2, Nanog and Notch1/2. 

## 5. Conclusions

Remodeling and polymerization of actin filaments and their associated actin-binding proteins are important in regulating tumor migration and invasion properties. Previous studies have suggested that Flii affects cSCC growth via its effects on tumor invasion and apoptosis and studies presented here demonstrate a role for Flii in regulating proteins involved in cSCC proliferation and tumor progression mediated by Wnt/β-catenin signaling. For the first time, we demonstrate a functional effect of Flii knockdown on cell division and differentiation of human aggressive metastatic cSCC cell line via its effects on the Wnt/β-catenin signaling pathway. Future studies could investigate the effect of Flii on factors that promote cSCC epithelial to mesenchymal transition, including the level of TGF and BMP at the tumor site [45] and Wnt5 and ROR2 expression at the leading edge of cSCC, which have been shown to regulate Snail-medicated EMT and invasive properties of cancer cells [47]. Additionally, this might shed light on the precise mechanism of action for Flii in cSCC metastasis. Taken together these studies suggests a potential role for Flii in aggressive metastatic cSCC, labeling Flii as a potential novel therapeutic target in aggressive cSCC. 

## Data Availability

Data are available upon request to the corresponding author.
